# Reduced Renal Mass, Salt-Sensitive Hypertension Is Resistant to Renal Denervation

**DOI:** 10.3389/fphys.2018.00455

**Published:** 2018-04-30

**Authors:** Ionut Tudorancea, Thomas E. Lohmeier, Barbara T. Alexander, Dragos Pieptu, Dragomir N. Serban, Radu Iliescu

**Affiliations:** ^1^Cardiology Division Department of Internal Medicine, Grigore T. Popa University of Medicine and Pharmacy, Iași, Romania; ^2^Department of Physiology, Grigore T. Popa University of Medicine and Pharmacy, Iași, Romania; ^3^CHRONEX-RD Biomedical Research Center, Grigore T. Popa University of Medicine and Pharmacy, Iași, Romania; ^4^Department of Physiology and Biophysics, University of Mississippi Medical Center, Jackson, MS, United States; ^5^Department of Plastic and Reconstructive Surgery, Grigore T. Popa University of Medicine and Pharmacy, Iași, Romania; ^6^Department of Pharmacology, Grigore T. Popa University of Medicine and Pharmacy, Iași, Romania; ^7^Regional Institute of Oncology, TRANSCEND Research Center, Iași, Romania

**Keywords:** blood pressure, salt-sensitive hypertension, renal disease, renal nerves, sympathetic nervous system

## Abstract

**Aim:** Activation of the sympathetic nervous system is common in resistant hypertension (RHT) and also in chronic kidney disease (CKD), a prevalent condition among resistant hypertensives. However, renal nerve ablation lowers blood pressure (BP) only in some patients with RHT. The influence of loss of nephrons *per se* on the antihypertensive response to renal denervation (RDNx) is unclear and was the focus of this study.

**Methods:** Systemic hemodynamics and sympathetically mediated low frequency oscillations of systolic BP were determined continuously from telemetrically acquired BP recordings in rats before and after surgical excision of ∼80% of renal mass and subsequent RDNx.

**Results:** After reduction of renal mass, rats fed a high salt (HS) diet showed sustained increases in mean arterial pressure (108 ± 3 mmHg to 128 ± 2 mmHg) and suppression of estimated sympathetic activity (∼15%), responses that did not occur with HS before renal ablation. After denervation of the remnant kidney, arterial pressure fell (to 104 ± 4 mmHg), estimated sympathetic activity and heart rate (HR) increased concomitantly, but these changes gradually returned to pre-denervation levels over 2 weeks of follow up. Subsequently, sympathoinhibition with clonidine did not alter arterial pressure while significantly suppressing estimated sympathetic activity and HR.

**Conclusion:** These results indicate that RDNx does not chronically lower arterial pressure in this model of salt-sensitive hypertension associated with substantial nephron loss, but without ischemia and increased sympathetic activity, thus providing further insight into conditions likely to impact the antihypertensive response to renal-specific sympathoinhibition in subjects with CKD.

## Introduction

The frequent association between chronic kidney disease (CKD) and treatment resistant hypertension (RHT) leads to a high incidence of adverse renal and cardiovascular outcomes, reflecting the likely, although not well studied, reciprocal potentiation of these conditions on the severity of the hypertension and its progression (Calhoun et al., 2008; De Beus et al., 2015; Rossignol et al., 2015; Thomas et al., 2016; Wolley and Stowasser, 2016). High levels of renal sympathetic nerve activity (RSNA), as found in many patients with RHT and normal kidney function (Grassi et al., 2015), may further diminish the excretory capacity of the injured kidneys and, therefore, exacerbate sodium retention, volume overload, and hypertension. While several studies have reported increased sympathetic activity to the skeletal muscle, RSNA has not been directly assessed in patients with CKD (Converse et al., 1992; Schlaich et al., 2009; Grassi et al., 2011, 2015; De Beus et al., 2014). Furthermore, although catheter-based renal denervation (RDNx) appears promising for the treatment of RHT, at present, the clinical results are inconclusive, revealing the need to better understand the determinants for a favorable blood pressure (BP) response to this novel treatment (Symplicity HTN-2 Investigators Esler et al., 2010; Bhatt et al., 2014; Persu et al., 2014; Iliescu et al., 2015). A particularly neglected area of investigation is the impact of CKD on the antihypertensive response to RDNx. Despite initial encouraging results from small-scale studies (Schlaich et al., 2013; Wallbach et al., 2014; Beige et al., 2015; Ott et al., 2015; Kiuchi et al., 2016; Hering et al., 2017), the efficacy and safety of RDNx in patients with RHT and CKD remain uncertain, as large clinical trials using this non-pharmacological approach for BP control have excluded patients with impaired renal function, for fear of worsening renal injury (Symplicity HTN-2 Investigators Esler et al., 2010; Bhatt et al., 2014; De Beus et al., 2014; Persu et al., 2014; Grassi et al., 2015; Wolley and Stowasser, 2016).

The overarching impetus for conducting the present study was to investigate the role of the renal sympathetic nerves in mediating the hypertension associated with reduced renal function. The remnant kidney model used in the present study has been the mainstay in the investigation of the pathogenesis of CKD and hypertension associated with reductions in functional nephron number (Koletsky, 1959; Brenner, 1985; Griffin et al., 1994; Ibrahim and Hostetter, 1998; Griffin et al., 2000; Hildebrandt et al., 2016). Surgical reduction of renal mass (RRM) by ∼80% (not to be confused with the model of renal infarction-induced hypertension caused by tying off branches of the renal artery) causes minimal injury to the remnant nephrons in the early stages of the hypertension (Koletsky, 1959; Griffin et al., 1994; Ibrahim and Hostetter, 1998; Griffin et al., 2000; Hildebrandt et al., 2016). Although the role of the sympathetic nervous system in mediating the hypertension in this model has not been established, the resulting phenotype of *salt-sensitive*, volume overload hypertension associated with loss of functional nephrons mimics the clinical situation of patients with CKD and RHT who commonly have inappropriately high levels of salt intake and hypertension that is frequently resistant to pharmacological therapy (Calhoun et al., 2008; Pimenta et al., 2009; Nerbass et al., 2015; Rossignol et al., 2015; Wolley and Stowasser, 2016). In contrast to the uncertainty regarding the role of the sympathetic nervous system in the RRM model of hypertension, there is a consensus that increased sympathetic activity likely contributes to the development of the *salt-insensitive* hypertension that follows infarction of two-thirds of the remnant kidney (Campese and Kogosov, 1995; Augustyniak et al., 2010; Veiga et al., 2016). In the infarction model of CKD, local ischemia has been postulated to trigger renal afferent sympathoexcitatory reflexes (De Beus et al., 2014; Grassi et al., 2015; Glassock and Rule, 2016). However, renal ischemia may not be uniformly present in patients with CKD (Michaely et al., 2012; De Beus et al., 2014; Glassock and Rule, 2016) and it is unclear whether reduced renal function itself may influence sympathetic activity. Therefore, from a mechanistic perspective, the RRM-salt model of hypertension used in the present study provides an untainted understanding of the fundamental impact of reduced baseline renal function *per se* on the antihypertensive response to RDNx in those instances in which there is limited renal parenchymal injury. Furthermore, a particular advantage of this model is that it is devoid of comorbidities (obesity, sleep apnea, hyperaldosteronism) and use of antihypertensive medications that alter sympathetic activity and, therefore, undoubtedly contribute to the variable BP response to RDNx in clinical trials.

Thus, we tested the hypothesis that regional-specific abrogation of sympathetic outflow to the kidneys by RDNx chronically lowers BP in this salt-sensitive model of hypertension. A unique experimental approach in this study was that BP was measured continuously over several weeks to allow precise longitudinal determinations of changes in BP throughout the development of the hypertension and, moreover, during the immediate and subsequent days after RDNx. Furthermore, from the analysis of sympathetically mediated BP oscillations, along with inhibition of central autonomic outflow with clonidine, we determined whether increased sympathetic drive to non-renal targets play a role in mediating the salt-sensitive hypertension. These approaches allowed for a mechanistic evaluation of the role of the sympathetic nervous system in mediating salt-induced hypertension associated with reductions in the number of functional nephrons.

## Materials and Methods

Eight adult male Sprague-Dawley rats (28–32 weeks of age) were obtained from the National Research Institute “Cantacuzino” (Bucharest, Romania). Rats were housed in a temperature (21–23°C) and humidity-controlled environment with a 12 h light/dark cycle, with *ad libitum* access to food and water, and were acclimatized for at least 3 weeks before the experimental protocols. Animals were fed a standard rodent diet containing either 0.8% NaCl (normal salt, NS), 0.1% NaCl (low salt, LS), or 4% NaCl (high salt, HS) (National Research Institute “Cantacuzino,” Bucharest, Romania). Surgical procedures were conducted under isoflurane anesthesia (2–3%). Tetracycline (2 mg/mL in drinking water) was administered for 3 days after each surgical procedure. All experiments were performed in accordance to the European Directive 2010/63/EU on the Protection of Animals Used for Scientific Purposes, the European Convention for the Protection of Vertebrate Animals used for Experimental and other Scientific Purposes (Council of Europe No. 123, Strasbourg, 1985) and the National Institutes of Health Guide for the Care and Use of Laboratory Animals and were approved by the Research Ethics Committee of the Grigore T. Popa University of Medicine and Pharmacy, Iași.

### Animal Preparation

#### Placement of Telemeters for Continuous BP Recording

For implantation of telemeters, incisions were made in the midline abdominal and left inguinal regions. The body of the telemetry transmitters (TRM54PB, Millar, Inc., Houston, TX, United States) was secured to the right flank of the inner abdominal wall with silk sutures (3-0; Ethicon, NJ, United States). A 2 cm piece of PE90 polyethylene tubing (Intramedic^TM^, Becton Dickinson, Sparks, MD, United states) was guided from the left iliac fossa through a 1 mm incision of the abdominal muscle layer toward the inguinal area. The solid-state pressure sensor was tunneled through the tubing (which was then removed) and a length of ∼3 cm was advanced into the femoral artery with the pressure sensing tip in the aorta, below the emergence of the renal arteries. The pressure sensor was then secured with silk threads placed around the femoral artery and at the emergence from the abdominal wall. Finally, the muscle layers were closed with silk sutures and the skin with stainless steel clips (Stoelting Co., Wood Dale, IL, United States).

#### Surgical Reduction of Renal Mass

The left and right kidneys were accessed via two longitudinal paravertebral incisions extending 1.5–2 cm caudally from the last rib. This retroperitoneal approach was chosen as it allows access to the kidney with minimal interference with the perirenal fat pads containing the renal nerve fibers. Following right nephrectomy, the upper and lower poles of the left kidney were cut with a scalpel blade so that a total of 75–80% of renal mass was removed. Hemostasis was obtained by gentle application of Gelaspon^®^ strips (Chauvin Ankerpharm GmbH, Berlin, Germany). Throughout the procedure care was taken to avoid any manipulation of the left renal hilum containing the renal nerves. Incisions were closed with silk sutures and wound clips after visual assessment to verify hemostasis.

#### Renal Denervation of the Remnant Kidney

The left kidney stump was accessed through a 1.5–2 cm ventral abdominal paramedian incision. Fibrous tissue and intestinal loops adherent to the remnant kidney were carefully removed using fine tweezers under magnification by a stereomicroscope (Zeiss, Jena, Germany). The renal artery and vein were exposed and cleared of surrounding tissue. All visible nerve running along the renal artery and vein within an area extending from the aorta to the renal hilum were cut. Thereafter, the adventitial layer was gently stripped off the renal vessels and a 10% phenol/ethanol (v/v) solution was applied for at least 5 min to the vessels using damp fine cotton tips. Extensive care was taken to prevent any leakage of the phenol solution onto the remnant kidney or into the peritoneal cavity. Finally, the surgical area was flushed with warm saline to remove any residual phenol. Wounds were closed with silk sutures and metal clips.

### Data Acquisition and Analysis

#### Continuous Recording of BP Waveform

Rats were placed on TR181 smartpads (Millar, Inc., Houston, TX, United States) for signal acquisition and wireless charging of the implanted telemeters. The individual 24-h BP waveforms were acquired continuously at a sampling frequency of 2000 Hz using a PowerLab 16/35 acquisition system (ADInstruments, Bella Vista, NSW, Australia) connected to a PC for storage and subsequent analysis. All individual cardiac cycles identified using LabChart^®^ built-in BP module (ADInstruments, Bella Vista, NSW, Australia) were averaged to compute mean arterial pressure (MAP) and heart rate (HR) every day from the continuous 7.00 AM to 6.00 AM recordings, excluding the 1 h necessary for daily animal care.

#### Estimation of Sympathetic Activity

Spectral analysis was performed using the Fast Fourier Transform, as we previously described (Iliescu et al., 2013). Briefly, the original daily BP signal sampled at 2000 Hz was analyzed in the frequency domain using the implementation of the LabChart^®^ software. Power spectra were calculated for all artifact-free segments ∼1 min in duration (131,072 data points) within every 23-h period, overlapping by 50% and windowed using a Hamming function, and finally averaged to yield daily BP spectra. Analysis of the direct BP signal was preferred over extraction of cardiac cycle-related variables such as the systolic BP as it avoided the inherent issues related to resampling for unequally spaced time-series. Since the frequency band between 0.25 and 0.75 Hz (low frequency, LF) contains BP oscillations originating from sympathetically driven variations in arterial vascular tone (Oliveira-Sales et al., 2014), the power in this band was integrated and expressed as percentage of the total power below the HR (0.01–3 Hz).

##### Pharmacological blockade

While on HS and after RRM and RDNx, global sympathetic activity was assessed from the hemodynamic and BP power spectral responses during administration of the centrally acting sympatholytic drug, clonidine. Clonidine (300–150 μg/kg/day; Sintofarm S.A., Bucharest, Romania) was administered in the drinking water for 6 and 3 days, respectively, in doses reported to reduce BP under conditions of sympathetic activation (Abdel-Rahman, 1994; Thomas et al., 2003; El-Mas and Abdel-Rahman, 2010). The lower dose was administered to avoid the potentially deleterious rebound effect from clonidine withdrawal. Fluid intake was monitored daily during clonidine administration and adjustments of the drug concentration were performed when necessary to achieve constant drug intakes.

### Experimental Design

After implantation of the telemeters, the rats were maintained on NS and monitored until the circadian rhythmicity of BP and HR was fully restored (10–14 days). Then, control hemodynamic variables were recorded while on NS. To assess the hemodynamic and neural responses to variations in salt-intake during normal renal function, the rats were then fed LS for 1 week, followed by 1 week of HS, before returning to LS. After 5 days on LS, the right kidney was removed and the poles of the left kidney were excised to produce a total reduction in renal mass of ∼75–80%. Then the rats were allowed to recover for 10 days before HS was initiated and maintained for 6 weeks. RDNx of the remnant kidney was performed after 2 weeks on HS, when BP levels were stable. Two weeks after RDNx, responses to clonidine were assessed. Finally, rats were again placed on LS until the end of the study. At weekly intervals throughout the study, the rats were placed in metabolism cages and urine was collected for 24 h in chilled containers at 4°C (Tecniplast S.p.A., Italy). Sodium concentration was determined in urine using standard techniques, as previously described (Hildebrandt et al., 2016). Urinary protein concentration was determined based on the absorbance at 280 nm, using bovine serum albumin as the standard (NanoDrop, Wilmington, DE, United States). At the end of the study, the remnant kidneys and normal kidneys from seven additional age-matched rats housed in similar conditions were harvested after cervical dislocation and immediately flash frozen in liquid nitrogen. Renal tissue norepinephrine content was measured by HPLC by the Hormone Assay and Analytical Services Core at Vanderbilt University Medical Center (Goldstein et al., 1981).

### Synopsis of Protocols

#### Normal Renal Function

Days 1–4, NS control

(1) Days 5–11, LS
(2) Days 12–18, HS
(3) Days 19–24, LS

#### Reduced Renal Mass

(1) Days 25–39, LS
(2) Days 40–53, HS
(3) Days 54–67, HS + RDNx
(4) Days 68–73, HS + RDNx + clonidine (300 μg/kg/day)
(5) Days 74–76, HS + RDNx + clonidine (150 μg/kg/day)
(6) Days 77–81, HS + RDNx (clonidine washout)
(7) Days 82–92, LS + RDNx

### Statistical Analysis

Results are expressed as mean ± SE. One-way repeated measures ANOVA followed by Dunnett’s multiple comparison test were used (Prism 6.01, GraphPad Software) to compare the following experimental periods.

#### Normal Renal Function

Daily hemodynamic responses during varying salt intake (Days 5–24) to NS control (Days 2–3); Weekly urine responses during varying salt intake to NS control.

#### Reduced Renal Mass

Daily hemodynamic responses during (1) HS (Days 40–53) to LS (Days 38–39); (2) HS + RDNx (Days 54–67) to HS (Days 52–53); (3) HS + RDNx + clonidine and clonidine washout (Days 68–81) to HS + RDNx (Days 66–67); and (4) LS (Days 82–92) to HS + RDNx (Days 80–81). Weekly urine responses during (1) HS, HS + RDNx, and HS + RDNx + clonidine to LS + RDNx and (2) HS, HS + RDNx, and HS + RDNx + clonidine to HS + RDNx (week 1). Differences were considered statistical significant for *P* < 0.05.

## Results

### Responses to Varying Salt Intake With Normal Renal Function

**FIGURE 1:**
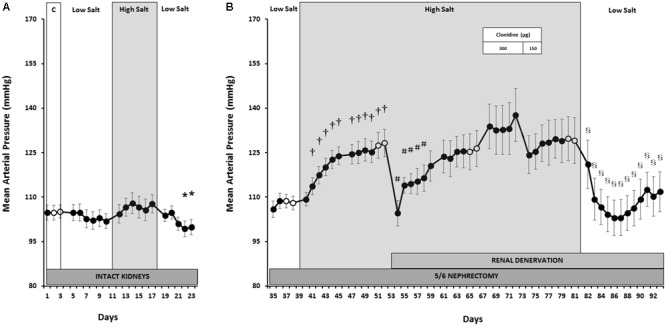
Mean arterial pressure responses to varying salt intake during normal renal function **(A)** and responses to RDNx and central sympathoinhibition during salt loading, RRM hypertension **(B)**. Values are mean ± SEM and *n* = 8. *Normal renal function*: ^∗^*P* < 0.05 vs. NS Control (Days 2–3). *Reduced renal mass*: ^†^*P* < 0.05 vs. baseline LS (Days 38–39); ^#^*P* < 0.05 vs. baseline HS (Days 52–53); ^§^ *P* < 0.05 vs. baseline clonidine washout (Days 80–81). Respective baseline days are depicted as (○).

**FIGURE 2:**
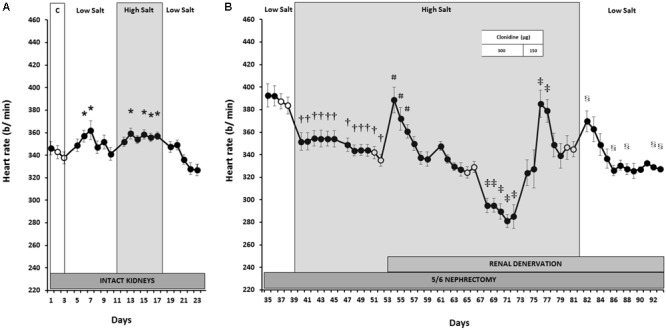
Heart rate responses to varying salt intake during normal renal function **(A)** and responses to RDNx and central sympathoinhibition during salt loading, RRM hypertension **(B)**. Values are mean ± SEM and *n* = 8. *Normal renal function*: ^∗^*P* < 0.05 vs. NS Control (Days 2–3). *Reduced renal mass*: ^†^*P* < 0.05 vs. baseline LS (Days 38–39); ^#^*P* < 0.05 vs. baseline HS (Days 52–53); ^‡^*P* < 0.05 vs. baseline HS + RDNx (Days 66–67); ^§^ *P* < 0.05 vs. baseline clonidine washout (Days 80–81). Respective baseline days are depicted as (○).

**FIGURE 3:**
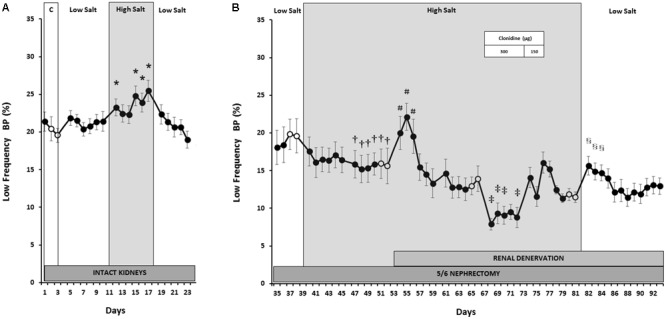
Estimated sympathetic activity (LF BP power) responses to varying salt intake during normal kidney function **(A)** and responses to RDNx and central sympathoinhibition during salt loading, RRM hypertension **(B)**. Values are mean ± SEM and *n* = 8. *Normal renal function*: ^∗^*P* < 0.05 vs. NS Control (Days 2–3). *Reduced renal mass*: ^†^*P* < 0.05 vs. baseline LS (Days 38–39); ^#^*P* < 0.05 vs. baseline HS (Days 52–53); ^‡^*P* < 0.05 vs. baseline HS + RDNx (Days 66–67); ^§^ *P* < 0.05 vs. baseline clonidine washout (Days 80–81). Respective baseline days are depicted as (○).

As shown in **Figure 1A**, MAP remained at NS control levels (105 ± 2 mmHg) during both LS and HS, although MAP was 5 mmHg below control during LS recovery. This indicates little or no BP salt sensitivity when renal function was normal. While transient increases in HR (**Figure 2A**) occurred during the initial days of LS, LF power (**Figure 3A**) remained unchanged from control levels for the entire week of LS. In contrast, HS led to significant and sustained increases of HR and estimated sympathetic activity as reflected by high levels of LF power. After returning the rats to LS in preparation for unilateral nephrectomy and partial surgical ablation of the remaining kidney, all variables returned to values indistinguishable from control except MAP, as indicated above. Urinary sodium and volume excretion closely paralleled the varying salt intakes while urinary protein excretion remained unchanged (**Table 1**).

**Table 1:** Weekly urinary responses during the different experimental periods.

	Intact kidneys			Reduced renal mass					
							Renal denervation		
		
	Normal salt control	Low salt	High salt	Low salt	High salt				
					Week 1	Week 2	Week l	Week 2	Clonidine
UNaV (mEq/day)	0.96 ± 0.1	0.35 ± 0.08	4.24 ± 0.8^∗^	0.41 ± 0.1	6.71 ± 1.3^#^	9.08 ± 0.6^#^	10.37 ± 0.9^#^	8.42 ± 0.7^#^	7.80 ± 1.0^#^
*V* (mL/day)	17.5 ± 1.40	16.8 ± 1.40	34.3 ± 4.20^∗^	29.6 ± 2.10	64.8 ± 6.30^#^	78.8 ± 4.40^#^	87.1 ± 2.70^†,#^	85.6 ± 5.60^†,#^	85.7 ± 3.50^†,#^
Proteinuria (mg/day)	12.5 ± 2.2	12.2 ± 2.3	15.88 ± 3.5	11.8 ± 1.9	17.6 ± 2.6	22 ± 4	22.3 ± 3.5	26.5 ± 7	24.9 ± 5.3

*Values are mean ± SEM (*n* = 8). UNaV indicates urinary sodium excretion. *V* indicates urine excretion. For intact kidneys, ^∗^*P* < 0.05 vs. NS Control. For reduced renal mass (RRM), ^#^*P* < 0.05 vs. LS + RRM; ^†^*P* < 0.05 vs. HS + RRM week 1.*

### Responses to Salt Loading After Reduction of Renal Mass

In contrast to the absence of changes in MAP with varying salt intakes when renal function was normal, after RRM MAP increased progressively during the first week of salt loading and plateaued during week 2 to levels ∼20 mmHg higher than LS baseline, clearly indicating salt sensitivity (**Figure 1B**). This hypertensive response was associated with a fall in HR (**Figure 2B**) and estimated sympathetic activity (**Figure 3B**), likely due to autonomic responses caused by pressure-induced baroreflex activation. Urinary sodium and volume excretion were significantly higher during the 2 weeks of HS compared to LS baseline while urinary protein excretion did not change significantly (**Table 1**).

### Responses to Renal Denervation After Salt-Induced Hypertension

During the first day after RDNx MAP fell dramatically by ∼24 mmHg, to levels similar to those recorded during LS intake (**Figure 1B**), while HR (**Figure 2B**) and estimated sympathetic activity (**Figure 3B**) increased concomitantly. However, these acute hemodynamic changes after RDNx were only transient as MAP recovered back to hypertensive levels, while HR and LF power decreased toward pre-denervation levels within a week and were stable thereafter. Urinary sodium and protein excretion remained at levels similar to the first week of HS + RRM while urinary volume excretion increased by ∼35% after RDNx (**Table 1**).

### Responses to Global Sympathoinhibition During Salt Loading Hypertension

As expected, administration of clonidine (300 μg/kg) led to sustained sympathoinhibition as reflected by marked suppression of sympathetically mediated oscillations of BP in the LF band (**Figure 3B**) and significant bradycardia (**Figure 2B**). However, MAP increased slightly (**Figure 1B**), albeit not significantly. During the 3 days of clonidine dose tapering (150 μg/kg) and the following washout period, estimated sympathetic activity gradually returned to control levels while MAP remained stable at post-denervation hypertensive values. Rebound tachycardia occurred for the first 2 days after cessation of clonidine, before eventual recovery by the end of the washout period. No further changes occurred in urinary variables during clonidine administration when compared to the days preceding drug treatment (**Table 1**).

### Responses to Salt Restriction After Reduced Renal Mass

During the initial 2 days of LS there were precipitous reductions in MAP (**Figure 1B**). Along with the abrupt fall in MAP, estimated sympathetic activity and HR increased during this time (**Figures 2B, 3B**). By the end of the LS recovery period, MAP and HR were significantly lower than the days preceding salt restriction while estimated sympathetic activity returned to baseline levels, once again showing salt sensitivity.

### Renal Tissue Levels of Norepinephrine

Renal levels of norepinephrine measured at the end of the experiment (∼6 weeks after RDNx) were 21.7 ± 5.9 ng/g, approximately 90% lower than those of age-matched normal animals (231.1 ± 26.1 ng/g, *P* < 0.001), indicating completeness of RDNx.

## Discussion

The major findings of the present study are: (1) RDNx lowered BP transiently but not chronically in this model of salt-sensitive hypertension, indicating that the renal nerves have little role in the long-term maintenance of the hypertension. (2) Global suppression of sympathetic activity with clonidine did not attenuate the hypertension. (3) Indirect measures of sympathetic activity are consistent with these BP responses in that they indicate that sympathetic activity is actually suppressed during RRM and HS.

Because there is independent activation of the sympathetic nervous system in many patients with CKD and RHT, sympathetic overactivity is believed to play a causative role in hypertension and disease progression (Converse et al., 1992; Schlaich et al., 2009; Grassi et al., 2011, 2015; De Beus et al., 2014). Therefore, inhibition of adrenergic drive is expected to be an effective therapy in these heterogeneous populations, especially when CKD and RHT coexist. However, not all patients with RHT have a satisfactory BP response to sympatholytic device-based therapies and the conditions for a favorable response are unclear, especially if CKD is present, as this has been an exclusion criterion in the large clinical trials (Symplicity HTN-2 Investigators Esler et al., 2010; Bisognano et al., 2011; Bhatt et al., 2014; Iliescu et al., 2014; Persu et al., 2014; Iliescu et al., 2015; De Leeuw et al., 2017). Furthermore, this understanding has been confounded by the multiple associated comorbidities and the variable antihypertensive medications given to these patients to control BP (Iliescu et al., 2015). These were non-confounders in the present study designed to evaluate the impact of renal-specific sympathoinhibition by RDNx in a model of salt-sensitive hypertension and CKD associated with loss of functional nephrons by surgical reduction of kidney mass.

Experimental studies suggest an important role of heightened sympathetic activity in the development of hypertension following infarction of two-thirds of the remnant kidney (Campese and Kogosov, 1995; Augustyniak et al., 2010; Veiga et al., 2016) and are consistent with the frequently invoked contention that activation of afferent sensory nerves originating in the injured kidney in CKD triggers reflex increases in sympathetic outflow (Schlaich et al., 2009; De Beus et al., 2014; Grassi et al., 2015). Renal ischemia is the mainstay of the infarction model and its putative role as the trigger for sympathoexcitatory renal reflexes is further supported by the finding that even milder restriction of blood supply to the kidneys, as found in renovascular hypertension, result in sympathetic activation (Oliveira-Sales et al., 2014). However, renal ischemia may not be uniformly associated with CKD (Michaely et al., 2012; De Beus et al., 2014; Glassock and Rule, 2016) and it is not clear whether hypertensive patients with reduced renal function not accompanied by ischemia have sympathetic activation and stand to benefit from RDNx. We used the surgical excision approach for RRM, which avoids direct injury to the remnant nephrons (Griffin et al., 1994) and denervated the remnant kidney *after* salt-sensitive hypertension had developed but within a time frame in which there is at best only moderate glomerulosclerosis (Koletsky, 1959; Griffin et al., 1994, 2000; Ibrahim and Hostetter, 1998). Thus, our data suggest that the condition of salt-sensitive hypertension associated with non-ischemic nephron loss may be a predictor of a “non-responder” phenotype to renal-specific sympathoinhibition by RDNx.

Because hypertensive patients and animals characterized by increased sympathetic activity have the most consistent antihypertensive response to RDNx, the most likely explanation for the failure of RDNx to chronically lower BP in the present study is the absence of sympathetic activation. In contrast to the findings in ischemic models of CKD, we found evidence of suppressed, rather than increased, estimated sympathetic outflow as LF of BP power and the HR actually decreased following RRM and HS. Sympathoinhibition may be due to sustained baroreflex activation during established salt-sensitive hypertension, as found in other models of hypertension (Lohmeier and Iliescu, 2015). Accordingly, we found no evidence for increased sympathetic activity in dogs subjected to the same model of RRM-salt-induced hypertension used in the present study (Hildebrandt et al., 2016). Furthermore, although we did not directly measure RSNA, the lack of sustained BP lowering after RDNx indicates that the prevailing level of renal sympathetic outflow makes a minor contribution, at best, to the maintenance of salt-sensitive hypertension in this nonischemic model of RRM. These results are consistent with findings in experimental studies in which RSNA is not elevated, studies reporting no sustained hypotensive response to RDNx in normotensive animals (Lohmeier et al., 2007; Kandlikar and Fink, 2011) and in animals in the early stages of salt-sensitive hypertension associated with mineralocorticoid excess and devoid of distinct renal parenchymal injury (Kandlikar and Fink, 2011; Lohmeier et al., 2015).

The hemodynamic responses on the days immediately following RDNx in the present study are consistent with the possibility that abolition of renal sympathetic outflow may still have acute effects to increase renal excretory function, even in the absence of overt sympathoexcitation. At that time, there was a pronounced fall in BP, consistent with increased fluid excretion and reduced extracellular fluid volume, although an effect of post-surgical stress could not be discounted. However, these hemodynamic responses did not persist chronically after RDNx. These acute and chronic changes in BP are consistent with observations in rats and dogs without increased RSNA subjected to RDNx and may reflect time-dependent antinatriuretic compensations that eventually oppose any expected sustained natriuretic effects due to loss of the tonic influence of the renal nerves on excretory function (Lohmeier et al., 2007; Kandlikar and Fink, 2011; Lohmeier and Iliescu, 2015). Thus, increased sympathetic activity appears to be an obligatory requirement for a long-term reduction in BP in response to RDNx.

Activation of renal afferent nerve fibers has been implicated in the sympathetic activation and hypertension associated with kidney disease (Schlaich et al., 2009; De Beus et al., 2014; Grassi et al., 2015). However, if RDNx had central sympathoinhibitory effects caused by interruption of afferent renal reflexes in the present study, vasodilatory and cardioinhibitory responses to sympathetic suppression would have occurred concomitantly with the acute reduction in BP after denervation. On the contrary, during the first days after RDNx, sympathetically driven oscillations in BP (as reflected by LF BP power) and HR increased markedly, presumably due to baroreceptor unloading. Thus, we found no evidence for a contribution of renal afferent reflexes to the modulation of sympathetic outflow in this model.

A number of studies have shown that salt-sensitive hypertension is associated with increased sodium concentration in plasma and/or cerebrospinal fluid, and acute studies report sympathetic activation occurs when similar increases in sodium concentration are achieved at these sites and in areas of the brain that are critical determinants of central sympathetic outflow (Stocker et al., 2013, 2015; Kinsman et al., 2017). Furthermore, several acute studies show that the increased central sympathetic outflow induced by increased sodium concentration is confined to non-renal districts (Stocker et al., 2013, 2015; Kinsman et al., 2017). Taken together, these acute studies support the contention that increases in peripheral resistance, and not reductions in renal excretory function, are causal in the genesis of salt-sensitive hypertension, a hypothesis not shared by many investigators, but nevertheless a recurring subject of debate (Hall, 2016; Kurtz et al., 2016). We therefore considered whether sympathetic activation to non-renal territories contributes to the hypertension in the absence of the renal nerves. Since we considered the possibility that the spectral analytical methods used may have been insufficiently sensitive to detect increases in global sympathetic activity after RDNx, we determined the sympathetic and hemodynamic responses to central sympathoinhibition by clonidine, administered chronically in doses that previously have been shown to lower BP and sympathetic activity in several experimental models of sympathetically mediated hypertension (Abdel-Rahman, 1994; Thomas et al., 2003; El-Mas and Abdel-Rahman, 2010).

As expected, clonidine clearly lowered post-denervation LF, indicating sustained sympathoinhibition. HR decreased as well but, most significantly, BP did not. This shows that in the absence of sympathoexcitation, global reductions in sympathetic activity to regions exclusive of the kidneys do not chronically lower BP. However, although we used doses of clonidine reported to reduce BP under conditions of sympathetic activation (Abdel-Rahman, 1994; Thomas et al., 2003; El-Mas and Abdel-Rahman, 2010) it should be noted that the central actions of clonidine to lower BP in the present study, in the absence of increased sympathetic activity, may have been masked by direct stimulation of vasoconstrictor peripheral alpha-2 adrenoreceptors (Francis, 1988; Lohmeier et al., 2009). These observations are especially relevant to clinical practice, as the BP response to clonidine has been proposed to determine the dependency of hypertension on elevated central sympathetic activity and predict the response to RDNx in RHT patients (Katholi et al., 2010). Taken together, these data indicate that in the non-ischemic CKD model of salt-sensitive hypertension, global sympathetic activity is suppressed, likely via a baroreflex-mediated mechanism, thus limiting the antihypertensive efficacy of sympatholytic approaches.

### Limitations

First, if progressive loss of function in hyperfiltering remnant nephrons were to lead to a time-dependent increase in BP, this could possibly account for the inability to demonstrate a sustained antihypertensive response to RDNx in the present study. However, this possibility is unlikely for the following reasons: (a) this study was performed during the early stage of salt-sensitive hypertension, below the time frame necessary for appreciable hypertensive nephrosclerosis in this model (Griffin et al., 1994, 2000; Ibrahim and Hostetter, 1998); (b) there is no progressive decrease in glomerular filtration rate in remnant nephrons over the time course of this study (Griffin et al., 1994); (c) urinary protein excretion was unchanged throughout the 5 weeks of HS; and (d) BP promptly returned to control levels at the end of the experiment when HS was replaced by LS. Thus, based on the above facts, it is unlikely that the addition of a sham control group for RDNx would have significantly altered interpretation of the current results or would have been justified, based on experimental, ethical, and financial considerations. Second, another issue common to all RDNx studies is verifying that the extent of denervation is sufficient to abolish the functional responses to activation of renal sympathetic nerves including during the chronic follow up period that may be associated with functional renal reinnervation. In the present study, these were unlikely cofounders as we used a standard surgical–pharmacological approach for extensive RDNx and showed that renal tissue norepinephrine levels at the end of the study were at an accepted level of suppression (<90% of control animals) for interruption of functional responses. Furthermore, and consistent with our findings, morphological evidence of nerve regrowth is not accompanied by an increase of renal norepinephrine content in rats, at least until 12 weeks post-denervation, well beyond the timeframe of our study (Rodionova et al., 2016).

## Conclusion

These findings in a classical model of kidney disease (Koletsky, 1959; Brenner, 1985; Griffin et al., 1994, 2000; Ibrahim and Hostetter, 1998; Hildebrandt et al., 2016) indicate that neither renal nor global sympathetic activation contribute to the salt-sensitive hypertension attributed solely to loss of functional nephrons. Furthermore, these data support the contention that increased sympathetic activity is an obligatory requirement for a sustained reduction in BP in response to RDNx. Since it is not feasible to expeditiously determine the level of sympathetic activation in a clinical setting, identification of the conditions responsible for sympathoactivation is crucial in the context of clinical trials showing that not all hypertensive patients have a favorable response to RDNx (Symplicity HTN-2 Investigators Esler et al., 2010; Bhatt et al., 2014; Persu et al., 2014; Iliescu et al., 2015). In this regard, a corollary to the present observations is that rather than simple reductions in renal function *per se*, signals from the injured kidney, such as ischemia, may underlie the reflex sympathetic activation, which promotes hypertension in CKD. Therefore, systematic exploration of the degree of ischemic renal injury may provide a mechanistic basis for identification of CKD patients with sympathetically mediated hypertension, which are likely to benefit the most from sympatholytic therapies such as RDNx.

## Author Contributions

IT designed and performed the experiments, analyzed the data, and drafted the manuscript. TL designed the experiments, performed the analytical measurements, analyzed the data, and reviewed the manuscript. BA performed the analytical measurements. DP designed the experiments, reviewed the manuscript, and provided the logistical support. DS designed the experiments, analyzed the data, reviewed the manuscript, and provided the logistical support. RI designed the experiments, performed the experiments, analyzed the data, ensured the funding, and drafted and reviewed the final manuscript.

## Conflict of Interest Statement

The authors declare that the research was conducted in the absence of any commercial or financial relationships that could be construed as a potential conflict of interest.
